# Association of Colecalciferol, Ferritin, and Anemia among Pregnant Women: Result from Cohort Study on Vitamin D Status and Its Impact during Pregnancy and Childhood in Indonesia

**DOI:** 10.1155/2018/2047981

**Published:** 2018-05-20

**Authors:** Raden Tina Dewi Judistiani, Lani Gumilang, Sefita Aryuti Nirmala, Setyorini Irianti, Deni Wirhana, Irman Permana, Liza Sofjan, Hesty Duhita, Lies Ani Tambunan, Jeffry Iman Gurnadi, Umar Seno, Reni Ghrahani, Agnes Rengga Indrati, Yunia Sribudiani, Tetty Yuniati, Budi Setiabudiawan

**Affiliations:** ^1^Public Health Department, Faculty of Medicine, Universitas Padjadjaran, Sumedang, West Java, Indonesia; ^2^Obstetric and Gynecology Department, Dr. Hasan Sadikin Hospital, Bandung, West Java, Indonesia; ^3^Obstetric and Gynecology Department, Waled Regency Public Hospital, Cirebon, West Java, Indonesia; ^4^Department of Child Health, Waled Regency Public Hospital, Cirebon, West Java, Indonesia; ^5^Obstetric and Gynecology Department, Syamsudin SH Public Hospital, Sukabumi, West Java, Indonesia; ^6^Obstetric and Gynecology Department, Cibabat General Hospital, Cimahi, West Java, Indonesia; ^7^Obstetric and Gynecology Department, Kota Bandung General Hospital, Bandung, West Java, Indonesia; ^8^Department of Child Health, Faculty of Medicine, Universitas Padjadjaran, Sumedang, West Java, Indonesia; ^9^Clinical Pathology Department, Faculty of Medicine, Universitas Padjadjaran, Sumedang, West Java, Indonesia; ^10^Department of Biochemistry and Molecular Biology, Faculty of Medicine, Universitas Padjadjaran, Sumedang, West Java, Indonesia

## Abstract

Studies had shown that iron-cycling was disturbed by inflammatory process through the role of hepcidin. Pregnancy is characterized by shifts of interleukin. Our objective was to determine if 25(OH) vitamin D (colecalciferol) status was associated with ferritin, anemia, and its changes during pregnancy.* Method.* A cohort study was done in 4 cities in West Java, Indonesia, beginning in July 2016. Subjects were followed up until third trimester. Examinations included were maternal ferritin, colecalciferol, and haemoglobin level.* Result.* 191 (95.5%) subjects had low colecalciferol, and 151 (75.5%) among them were at deficient state. Anemia is found in 15 (7.5%) subjects, much lower than previous report. Proportion of anemia increased by trimester among women with colecalciferol deficiency. Ferritin status and prepregnancy body mass index in the first trimester were correlated with anemia (*r* = 0.147, *p* = 0.038 and *r* = −0.56, *p* = 0.03). Anemia in the second trimester was strongly correlated with anemia in the third trimester (*r* = 0.676, *p* < 0.01).* Conclusion.* Our study showed that the state of colecalciferol was not associated with either ferritin state or anemia, but proportion of anemia tends to increase by trimester in the colecalciferol deficient subjects.

## 1. Introduction

The emerging awareness on hypovitaminosis D as major health problem across countries and regions has driven more researches from observational studies to clinical trials. Hypovitaminosis D, defined by serum 25-hydroxy vitamin D (colecalciferol) level below 25 nmol/L, is most common in South Asia and the Middle East [1]. Other studies classified the state as deficient, insufficient, and normal with respect to the disease of interest and also geographical area [1–8]. Certain characteristics were linked to hypovitaminosis D such as life style, less sunlight exposure, dietary habits, and not having fortified food, all included in the major factors that were significantly associated with lower colecalciferol levels [1]. The first report of colecalciferol status among pregnant women from a study in West Java, Indonesia, year 2016, stated that only 4.4% of 160 pregnant women in their first trimester had normal level of colecalciferol (>30 ng/mL), approximately 70% were insufficient (20–29 ng/mL), and the remaining 25.6% were deficient (<20 ng/mL) [9].

Anemia is also a global health problem not only for its prevalence but also for the burden caused by anemia itself. Kassebaum reported that global anemia prevalence in 2010 was 32.9%, accounted for 8.8% of the total disability from all condition; children and women were more affected [10]. Studies also reported that maternal anemia is associated with fetal and neonatal well-being [11–13]. The latest study on anemia among pregnant women in Jatinangor-West Java was as high as 21.9% [14].

Pregnancies are characterized by a shift toward proinflammatory mediators in later gestational age, while the opposite condition occurs in the earlier period to avoid pregnancy failure [15–18]. The link between anemia and inflammation was found from the study by Nicolas through the gene encoding hepcidin [19]. Later studies had proven that hepcidin was the king regulatory for iron hemostasis, allowing transfer of iron stores to blood circulation [20–23].

The clinical importance of colecalciferol deficiency with regard to anemia among pregnant women in Indonesia had never been reported until this article was written. We suspected that there might be an association of colecalciferol and haemoglobin. With iron as a crucial component for haemoglobin production, we know that the ultimate regulation of iron metabolism in human body is by the role of hepcidin, in the hepcidin-ferroportin gate system [22]. One study by Baccheta stated that hepcidin is downregulated by colecalciferol, but the study by Koenig found that different state of pregnancy may also influence the hepcidin level [21, 24]. In the iron-regulatory process ferritin has been formerly known as one of indicators for iron store besides transferrin. In absolute iron deficiency, low serum ferritin level tends to reflect low iron reserves, but other evaluations has been suggested like transferrin saturation to exclude iron depletion of other causes [25]. With its iron component, ferritin also can act as an acute phase reactant to inflammatory process so that a single measurement of normal or high ferritin may undermined the presence of actual iron deficiency [25].

The importance of assuring iron stores is mainly related to prevent of anemia, but the cause of anemia itself is rarely in isolation [26]. While clinical significance of ferritin evaluation should be the main focus for health service, knowledge development on iron metabolism is of equal importance to support treatment. Colecalciferol and the hepcidin-ferroportin iron-regulatory axis may be altered in pregnancy because pregnancy itself is a proinflammatory condition; therefore some changes in ferritin and transferrin level might occur according to the level of colecalciferol. This study aimed at finding out if colecalciferol and serum ferritin level in the first trimester would have an association with haemoglobin level or anemia in pregnancy.

The prevalence of anemia among pregnant in Indonesia has been declining over the period of 1997 to 2008, yet the last figure we found was as high as 35% or more, despite iron supplementation implementation of antenatal care policy during that period [27]. Regional areas in Indonesia had varied figures, perhaps due to chronic infection of tuberculosis, malaria endemicity, and malnutrition and it might also be due to different tools used for screening. One study which was also conducted in a small area of West Java showed approximately 65% difference in prevalence of anemia among pregnant women, using capillary finger prick test versus complete blood count from vein [14]. It was very likely that the correct prevalence of anemia among pregnant women in that study population was 21%, so we used this figure to compare our result [14].

The definition of anemia in pregnancy recommended by the Centre for Disease Control and Prevention is a haemoglobin (Hgb) or haematocrit (Hct) value less than the fifth percentile of the distribution of Hgb or Hct in a healthy reference population based on the stage of pregnancy [28]. As not every country has its own database, we refer to classification of anemia which was derived from an iron-supplemented pregnant women population, based on Hgb level and trimesters as follows: (1) Hgb levels below 11 g/dl in the first trimester; (2) Hgb below 10.5 g/dl in the second trimester; and (3) Hgb below 11 g/dl in the third trimester [28].

## 2. Material and Methods

This report was a part of cohort study on vitamin D status and its impact during pregnancy and childhood in Indonesia, with special interest in colecalciferol, nutrition, immunological changes and their association with both maternal and fetal outcomes. The cohort started in July 2016 in Bandung, Cimahi, Sukabumi, and Waled, West Java, Indonesia [9]. We expected to include 300 subjects to fit our budget, with approximately equal number from all cities. Our study protocol had been reviewed and ethical clearance was released from Health Research Ethical Committee of Faculty of Medicine, Universitas Padjadjaran.

### 2.1. Inclusion of Study Subjects

Prior to recruitment, pregnant women were given information about the study by community midwife at the primary health centres of associated sites. If they were interested, they were referred to study site hospitals, that is, Dr. Hasan Sadikin Hospital and Kota Bandung Hospital (Bandung sites), Cibabat Hospital (Cimahi site), Samsudin Hospital (Sukabumi site), and Waled Hospital (Waled site). At these hospitals complete information regarding this study was given by our trained midwives in the research team. After giving written consent, further obstetric anamnesis or confirmation and ultrasound screening were performed by our participating obstetricians. These obstetricians had followed training for standard setting in ultrasound examinations. Eligible subjects were women with singleton pregnancy between 10 and 14 weeks, fetus was normal, and subjects would comply with our study procedures. Recruitment was conducted consecutively until the expected numbers 70–75 were met at each site.

### 2.2. Steps after Recruitment

Each pregnant woman was interviewed for personal data, obstetric history, and knowledge on nutrition support in pregnancy by research team midwives. Pregnant women were then taught to fill in a three-day diary within the following week. The diary was about the food they consumed, how they dressed, and their activity pertaining to duration of sun exposure. We also set to repeat obstetric ultrasound examination, selected interview, and laboratory tests in the second and third trimester follow-up visits. All women were given freedom to choose their birth attendants and sites of baby deliveries and during labor they were also accompanied by our team midwives who would observe, but not interfere with, the labor process. Our team recorded any events until completion of stage 4 of labor. All records of each pregnant woman were kept as individual case report file in data storage for later entry.

### 2.3. Collection, Preservation, and Transfer of Blood Samples for Examination of Haemoglobin, Vitamin D, and Ferritin Level

Blood drawn from median cubital veins were directly transported to local site hospital lab for complete blood count and serum preparation. The separated serum was stored at −20 degree Celsius, before being transported to Dr. Hasan Sadikin Hospital in coolbox for colecalciferol and ferritin analysis. The longest transport time was from Waled and Sukabumi, which were up to 4 hours.

### 2.4. Complete Blood Count, Vitamin D, and Ferritin Assay and Interpretation

All complete blood count measurements were done using automated hematology analyzer with impedance method measurement (Sysmex XP-100, Japan). It was used to measure at least 8 parameters including Hb, mean corpuscular volume (MCV), and red blood count (RBC). Vitamin D and ferritin measurement was performed by ELISA.

Anemia was defined by Hgb level and trimesters as follows: (1) Hgb levels below 11 g/dl in the first trimester; (2) Hgb below 10.5 g/dl in the second trimester; and (3) Hgb below 11 g/dl in the third trimester. We also calculated Mentzer index to detect any pregnant women of *β* thalassemia trait and excluded one case.

The minimum measurable level of vitamin D in serum was 8.1 ng/mL; any level below that was reported as 8 ng/mL. Vitamin D status was defined as (1) deficient if its level was below 20 ng/mL; (2) insufficient if its level was between 20 and 29.99 ng/mL; and (3) normal if its level was 30 ng/mL or more.

Pregnant women with low iron store (hypoferritinemia) were defined if serum ferritin was below 30 ng/mL and normal if 30 ng/mL or more.

### 2.5. Statistical Analysis

Three members of our team performed entry and data cleaning from case report file into our customized Excel sheet (Microsoft Corp., USA). Making sure that data was cleaned, it was transferred into SPSS software. Only data on pregnant women characteristics, haemoglobin, and ferritin laboratory results were used for this article. The remaining data would be reported in our next report. Descriptive analysis on characteristics and laboratory data were done; dispersion analysis was conducted by Kolmogorov-Smirnoff and resulted in the fact that our data showed skewness (test result not shown). We therefore performed two sets of nonparametric analysis.

## 3. Result and Discussion

Between July 2016 and July 2017 a total of 201 pregnant women had been completely followed up until third trimester and included for this report. The others were recruited later that they had not met our minimum observation time. Only one subject had a low Mentzer index (<13) and anemia; she was excluded from analysis. The characteristics of our study subjects were as shown in Table 1.

In this study the terminology of “at risk” was chosen instead of “low risk” due to our own perspective that no single pregnancy is risk-free and that the switch from normal to pathologic conditions could happen in an instant. Our study population had high-risk cases, that is, 45 cases form age group (<20 years or >35 years) and 79 cases from parity group (parity 4 or more). The previous study had lower proportion of high-risk group on parity (11% versus 39.8%) but there were no data on maternal age and prepregnancy BMI [14].

The nutritional state was defined by prepregnancy body mass index classification from WHO [29]. The result showed that about 27% of our subjects had nutritional problem in the extremes. There were more underweight women than obese which could be a risk factor the presence of anemia.

We recruited pregnant women from first trimester, while in previous study we recruited women from both first and second trimester [14]. First trimester laboratory results from all 200 study subjects are presented in Table 2. Colecalciferol and ferritin level in the second and trimester were not available, yet by the time this article was written.

The number of study subjects who had returned for follow-up visit in the second and third trimester was lower than in first trimester for several reasons, some just failed to show up, and others were not yet due to schedule. The laboratory results from our study showed that 193 subjects (96.5%) had hypovitaminosis D (below 30 ng/mL); that is, 151 (75.5%) were deficient and 42 (21%) were insufficient in the first trimester. One study in the United States reported that their prevalence on hypovitaminosis D among pregnant women in their first trimester reached 70%, with mean serum concentration of 27.6 ng/mL and range of 13–71.6 ng/mL [30]. Mean colecalciferol from our study result was much lower, even below the cutoff for colecalciferol deficiency (Table 2). A different result came from a study in Tunisia which reported that no mother had an adequate vitamin D status during term delivery, with so much lower mean serum colecalciferol concentrations at 6.82 ± 5.14 ng/mL (range 3.60–23.77) [31].

**Figure 1:**
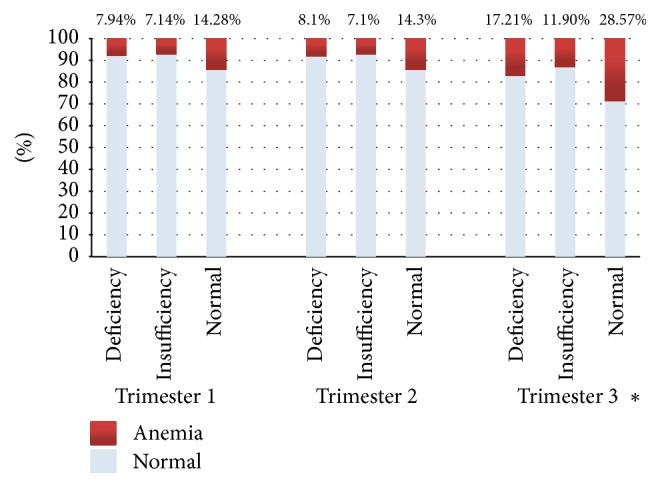
Proportion of subjects with anemia in trimesters 1, 2, and 3 by first trimester colecalciferol status. ^*∗*^Relative risk (95% CI) for anemia in the third trimester among subjects with calciferol deficiency and insufficiency in the first trimester, combined = 2.96 (0.36–24.63).

Figure 1 showed that subjects who were deficient or insufficient of colecalciferol state in the first trimester had higher risk to develop anemia in the third trimester, RR (95% CI) = 2.96 (0.36–24.63), and that the proportion of subjects with anemia increased in every subgroup colecalciferol state.

Anemia was present in 15 (7.5%) subjects of our study. This figure was much lower compared to two previous studies in Indonesia. Susanti, whose study was more similar to ours, reported a 21% prevalence of anemia in pregnancy in 2017, while Barkley reported that anemia in pregnant women above 15 years old was as high as 37.3% as a result from Indonesian Family Survey [14, 27]. Twenty-four point nine percent of our study subjects had hypoferritinemia (below 30 ng/mL) in the first trimester. We had not found any report which specifically reported on the actual cutoff of serum ferritin level to assess iron deficiency among pregnant women in Indonesia. A distinction between absolute and functional iron deficiency was also very important in pregnancy, even more with those who had chronic inflammation.

**Figure 2:**
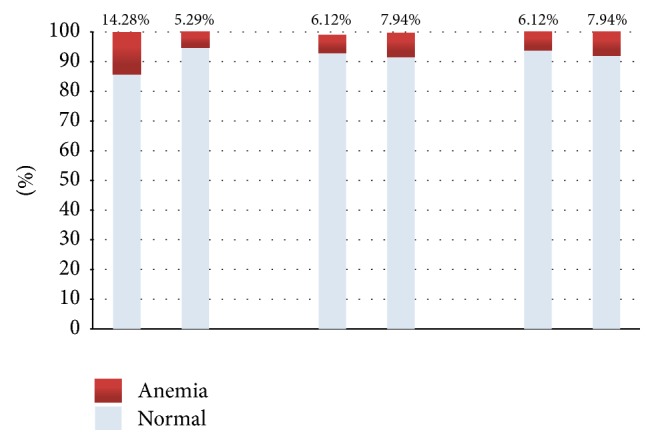
Proportion of subjects with anemia in trimesters 1, 2, and 3 by first trimester ferritin status. Correlation of first trimester ferritin level and first trimester anemia (*r* = 0.147, *p* = 0.038), no correlation found with second or third trimester anemia.

Figure 2 showed the comparison of anemia in trimesters 1, 2, and 3 based on the state of ferritin level in the first trimester only.

Our study showed that first trimester ferritin status was correlated positively with the presence of anemia in the first trimester, but not with the second or third trimesters. One report showed that in spite of satisfactory iron reserves with normal or even increased serum ferritin, the availability of iron for the bone marrow is limited substantially due to increased hepcidin transcription in chronic inflammation [32]. Unfortunately the examination to exclude chronic inflammation has not become a routine part in prepregnancy assessment. It is especially important as Indonesia is known to be one of the countries with highest tuberculosis burden [33]. Tuberculosis screening is not provided in routine prenatally for its high cost.

We calculated and found no correlation between age, parity, and BMI class at baseline and the state of colecalciferol and serum ferritin, so the statistical analysis results are not shown.

As we recruited pregnant women from first trimester, we observed that the number of anemia cases showed quite some changes from one trimester to another and from one state to another, especially with regard to colecalciferol deficiency. Haemodilution is known as the main factor for the presence of anemia in the second trimester, which may explain why the proportion of anemia was higher than in first trimester. Further exploration turned that haemoglobin level dropped even further among all of subjects with anemia in the second trimester, in average −1.44 gr/dL. Fifteen subjects who were normal in second trimester also had dropped in average −0.99 dr/dL. These findings needed more exploration on the iron-cycle itself during pregnancy. It was quite an important alarm as we know that the presence of anemia near parturition or at parturition also increased the risk for postpartum hemorrhage.

## 4. Conclusion

Our study showed that first trimester state of colecalciferol was not associated with ferritin. Subjects with vitamin D deficiency in the first trimester were more prone to develop anemia, with increasing proportion as pregnancy progressed to third trimester. First trimester ferritin state was correlated with anemia in the first trimester.

## Figures and Tables

**Table 1:** Descriptive summary of study subjects' characteristic.

Characteristics group	Number (%)	Mean (+SD)	Median ( IQR)
Age (years)		28.38 (±5.9)	28.0 (16–43)
At risk	155 (77.61)		
High risk	45 (22.39)		
BMI (kg/m^2^)		23.27 (±5.44)	22.04 (14.67–50.89)
Underweight	32 (16.92)		
Normal	112 (56.21)		
Overweight	34 (16.91)		
Obese	21 (10.44)		
Parity		na	na
At risk	121 (60.20)		
High risk	79 (39.80)		

*Note*. 1 case of suspected *β* thalassemia trait was excluded from analysis; na = not applicable.

**Table 2:** Descriptive summary of laboratory results in trimester 1.

	Mean (+standard deviation)	Median (interquartile range)
Colecalciferol (ng/mL)	15.34 (+6.99)	14.25 (8.0–43.5)
Ferritin (ng/mL)	67.81 (+53.81)	50.22 (4.8–306)
Haemoglobin (gr/dL)	12.58 (+1.18)	12.80 (8.2–14.4)
